# Beyond the Midline: An Unusual Presentation of Acanthomatous Ameloblastoma in the Anterior Maxilla

**DOI:** 10.7759/cureus.115805

**Published:** 2026-09-04

**Authors:** Vedika V Pillai, Shams Ul Nisa, Anshuman Jamdade, Amit A Mhapuskar, Jikisha Jain

**Affiliations:** 1 Oral Medicine and Radiology, Bharati Vidyapeeth (Deemed to Be) University Dental College and Hospital, Pune, IND; 2 Oral Medicine and Radiology, Mahatma Gandhi Dental College and Hospital, Jaipur, IND; 3 Oral and Maxillofacial Surgery, Bharati Vidyapeeth (Deemed to Be) University Dental College and Hospital, Pune, IND

**Keywords:** acanthomatous ameloblastoma, anterior maxilla, cone-beam computed tomography, odontogenic tumour, partial maxillectomy

## Abstract

Acanthomatous ameloblastoma is an uncommon histopathological variant of ameloblastoma characterised by extensive squamous metaplasia and keratin formation within odontogenic epithelial islands. It predominantly affects adults between 30 and 60 years of age and most commonly occurs in the posterior mandible; involvement of the anterior maxilla is rare. A 55-year-old man presented with a progressively enlarging swelling in the anterior maxilla accompanied by pain during mastication. Intraoral examination revealed a diffuse swelling that crossed the midline, involved the labial vestibule, and caused expansion of the labial cortical plate. Cone-beam computed tomography demonstrated a multilocular lesion with cortical perforation, external root resorption, tooth displacement, and involvement of the nasopalatine canal. Histopathological examination confirmed acanthomatous ameloblastoma. The patient underwent partial maxillectomy with complete surgical excision. Postoperative healing was uneventful, and no clinical or radiographic evidence of recurrence was detected during two years of follow-up. This case highlights the importance of correlating clinical, radiographic, and histopathological findings for accurate diagnosis and appropriate management of maxillary ameloblastoma. Long-term surveillance remains essential because of its locally aggressive behaviour and potential for late recurrence.

## Introduction

Ameloblastoma is a benign but locally aggressive odontogenic epithelial neoplasm arising from remnants of the dental lamina, enamel organ, or odontogenic epithelium. It accounts for approximately 10% of odontogenic tumours and occurs predominantly in the mandible, while maxillary involvement is relatively uncommon [1].

Acanthomatous ameloblastoma is an uncommon histopathological variant characterised by extensive squamous metaplasia and keratin formation within the stellate reticulum-like cells of odontogenic epithelial islands. Although its biological behaviour resembles that of conventional ameloblastoma, its unusual histopathological appearance may complicate diagnosis by mimicking other odontogenic and non-odontogenic lesions [2,3]. Accurate diagnosis therefore requires correlation of clinical, radiographic, and histopathological findings. Cone-beam computed tomography (CBCT) is particularly valuable because it provides high-resolution three-dimensional assessment of lesion extent, cortical perforation, root resorption, tooth displacement, and involvement of adjacent structures [4].

Maxillary ameloblastomas are clinically important because the thin maxillary cortices and proximity to the nasal cavity, maxillary sinus, orbit, and skull base may permit considerable extension before obvious external signs develop. An anterior maxillary lesion that crosses the midline is particularly unusual and may resemble other expansile odontogenic lesions. The present report is therefore noteworthy for the uncommon anterior maxillary location, midline-crossing growth, cortical perforation, root resorption, tooth displacement, and nasopalatine canal involvement. It illustrates how clinical examination, three-dimensional imaging, and histopathology complement one another in establishing the diagnosis and planning treatment [5,6].

## Case presentation

Case presentation

The patient was evaluated in the Department of Oral Medicine and Radiology and was subsequently diagnosed with acanthomatous ameloblastoma of the anterior maxilla. The case protocol was approved by the Institutional Ethics Committee (Approval No. EC/NEW/INST/2021/MH/0029). Written informed consent was obtained from the patient for the diagnostic and therapeutic procedures and for the publication of anonymised clinical information, radiographs, histopathological findings, and clinical photographs. This report was prepared in accordance with the CARE case-reporting guidelines [7].

Patient information

A 55-year-old man presented with a progressively enlarging swelling in the anterior maxilla accompanied by intermittent pain during mastication. The patient reported gradual progression; however, the exact duration from first noticing the swelling to presentation was not available in the clinical record. His medical history was unremarkable, with no systemic illness, previous surgery, or long-term medication use. There was no family history of odontogenic tumours or other hereditary disorders, and no relevant adverse habits were reported.

Clinical findings

The patient was conscious, cooperative, moderately built, and systemically healthy, with normal vital signs. Extraoral examination revealed no facial asymmetry (Figure 1a). The overlying skin appeared normal, without ulceration, discolouration, or sinus formation, and no cervical lymphadenopathy was detected.

Intraoral examination revealed a solitary, diffuse swelling involving the labial alveolar mucosa of the anterior maxilla. Anteroposteriorly, the swelling extended from the mesial aspect of tooth 21 to the distal aspect of tooth 13; superoinferiorly, it extended from the labial vestibule to the alveolar region of teeth 11-23. The clinically apparent swelling measured approximately 5 × 6 cm at its greatest dimensions. The overlying mucosa appeared stretched and showed prominent reddish-blue discolouration, with expansion of the labial cortical plate (Figure 1b). On palpation, the swelling was bony hard and tender, with areas of eggshell crackling. Tooth 11 had previously undergone inadequate endodontic treatment. The clinical measurement represents the overall visible and palpable swelling and should not be interpreted as directly equivalent to the smaller osseous dimensions measured on CBCT. 

**Figure 1:**
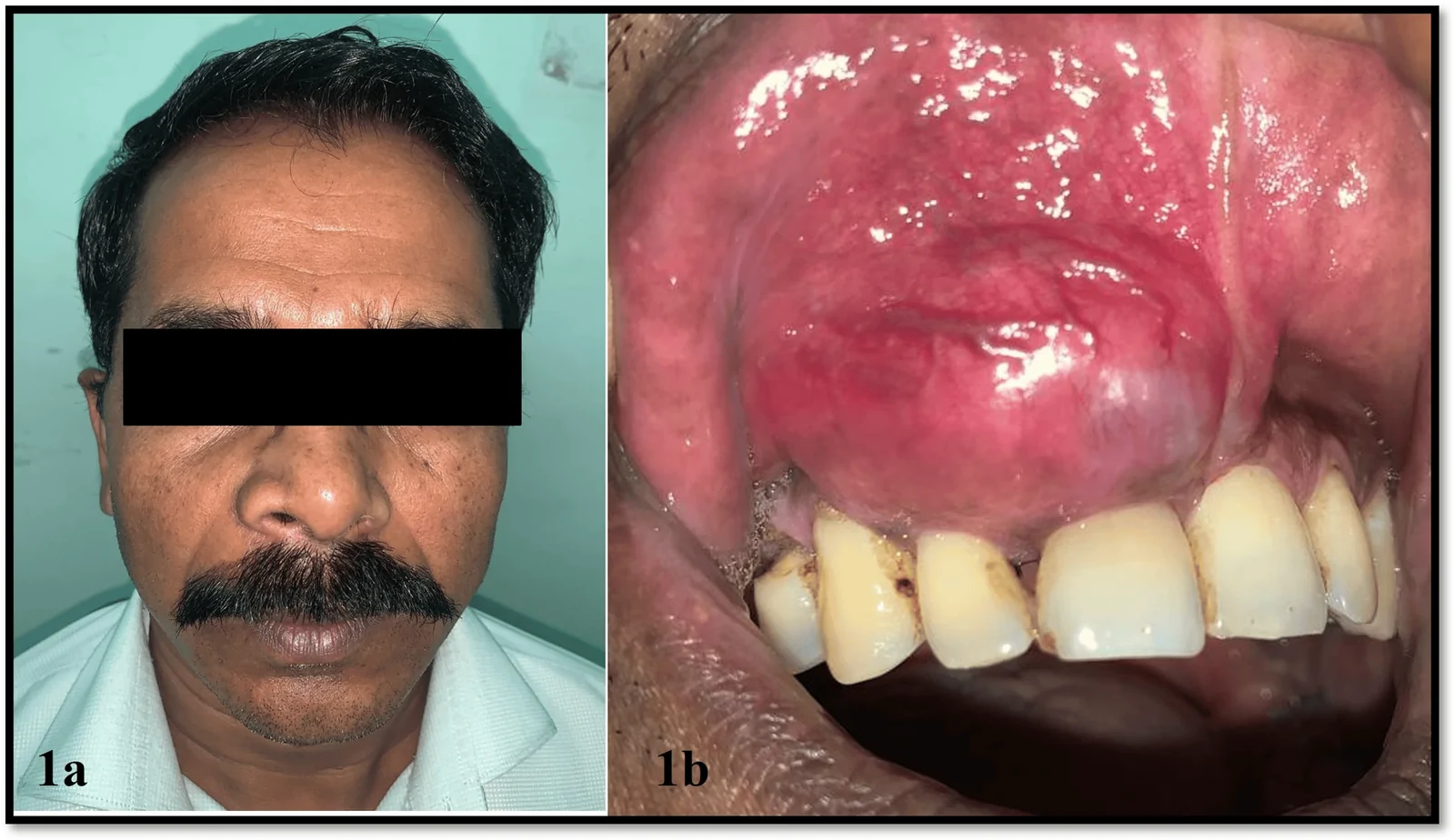
(a) Extraoral photograph showing no facial asymmetry. (b) Intraoral photograph showing a diffuse swelling involving the labial alveolar mucosa of the anterior maxilla, crossing the midline and exhibiting focal erythematous and bluish discoloration. Written informed consent was obtained from the patient for the publication of this image.

Diagnostic assessment

The clinical and radiographic presentation was diagnostically challenging because several benign odontogenic lesions can produce similar findings, including cortical expansion, root resorption, and tooth displacement. Based on the clinical findings, an aggressive benign odontogenic tumour was provisionally diagnosed.

Panoramic radiography demonstrated a mixed radiolucent-radiopaque lesion in the anterior maxilla, with displacement of adjacent teeth and external root resorption (Figure 2a). CBCT revealed a multilocular hypodense lesion involving the anterior maxilla. Superoinferiorly, the lesion extended from the right nasal floor to the alveolar crest; mesiodistally, it extended from the distal aspect of tooth 21 to the mesiobuccal root of tooth 16. It perforated the labial cortical plate, thinned the lateral wall of the nasal cavity, displaced the nasal floor superiorly, and involved the nasopalatine canal. External root resorption affected the adjacent teeth, particularly teeth 14 and 15, and tooth 11 was displaced inferiorly. Irregular internal septa produced a honeycomb appearance (Figure 2b-e). The CBCT measurement of approximately 26.2 × 25.3 mm refers to the measurable intraosseous component on the displayed sections, whereas the 5 × 6 cm clinical estimate described the broader visible and palpable swelling. Based on these findings, a radiographic diagnosis of ameloblastoma was made. Odontogenic keratocyst and central giant cell granuloma were considered in the differential diagnosis.

**Figure 2:**
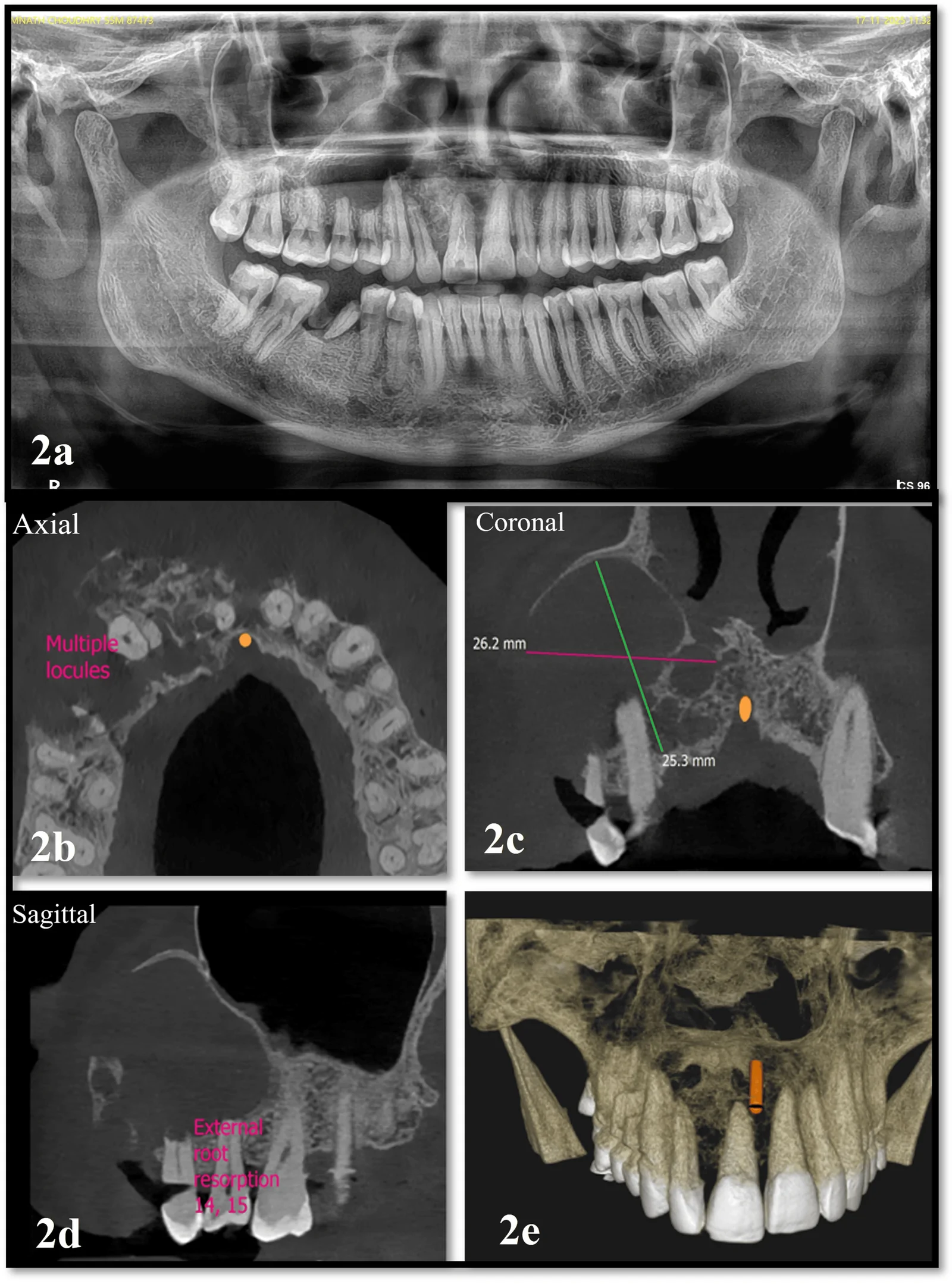
(a) Panoramic radiograph demonstrating an expansile mixed lesion in the anterior maxilla, with displacement of adjacent teeth and external root resorption. (b-e) Axial, coronal, sagittal, and three-dimensional CBCT reconstructions demonstrating a multilocular hypodense lesion with a honeycomb appearance, measuring approximately 26.2 × 25.3 mm at its greatest dimensions. CBCT: cone-beam computed tomography.

An incisional biopsy confirmed acanthomatous ameloblastoma. Histopathological examination demonstrated odontogenic epithelial islands with peripheral palisading of ameloblast-like cells, reverse nuclear polarity, central stellate reticulum-like areas, and extensive squamous metaplasia with keratin formation (Figure 3a,b). Representative microscopic features are indicated by arrows in Figure 3b.

**Figure 3:**
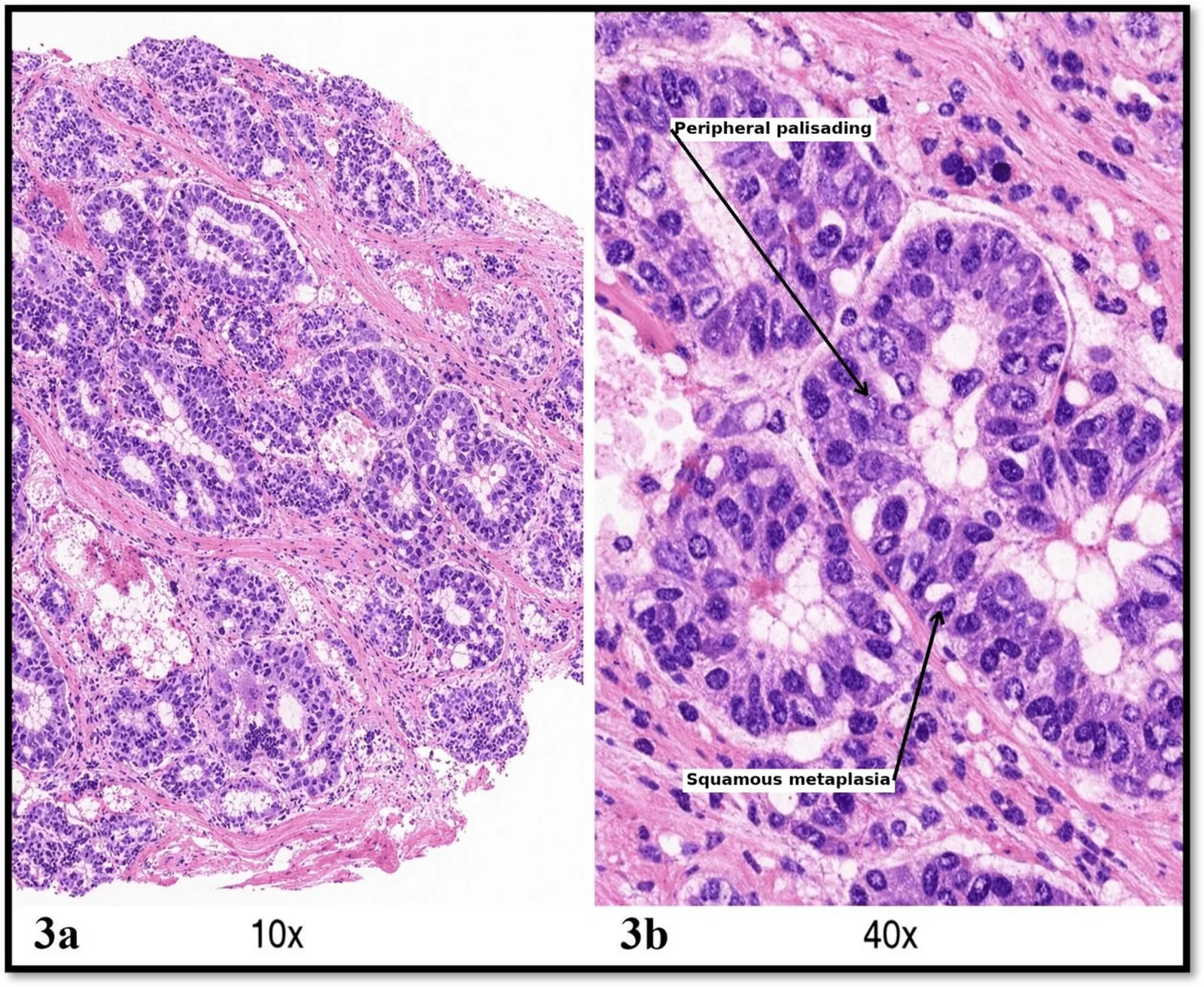
Hematoxylin and eosin-stained photomicrographs showing the characteristic histopathological features of acanthomatous ameloblastoma. (a) Low-power view (×10) showing multiple odontogenic epithelial islands within a fibrous connective tissue stroma. (b) High-power view (×40) showing peripheral palisading of ameloblast-like cells with reverse nuclear polarity and central squamous metaplasia with keratin formation. Arrows indicate the representative microscopic features.

Treatment

Given the locally aggressive nature and extent of the lesion, partial maxillectomy was performed under general anaesthesia, and the tumour was completely excised (Figure 4a,b). The resected specimen was submitted for histopathological examination, which confirmed the diagnosis of acanthomatous ameloblastoma.

**Figure 4:**
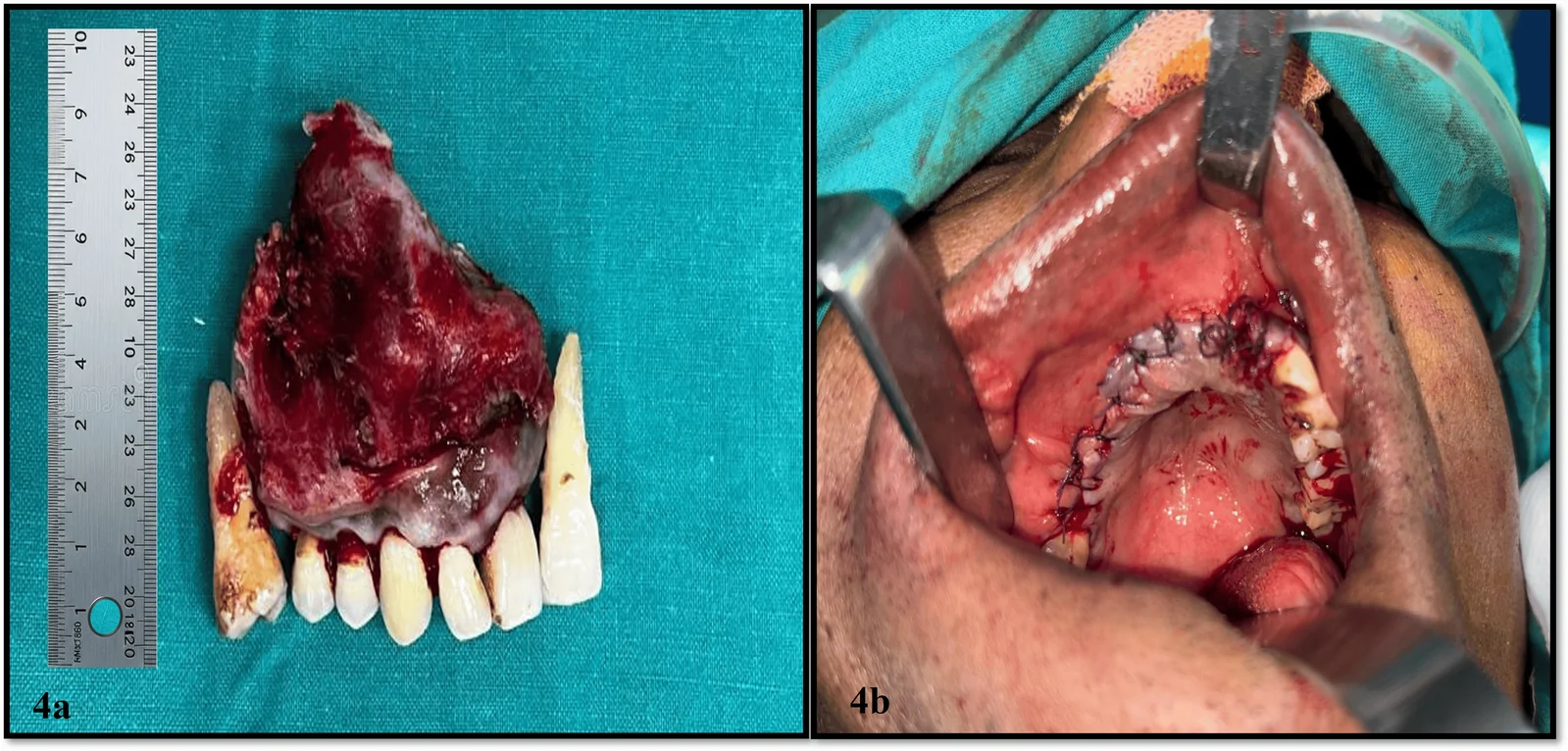
(a) Resected partial maxillectomy specimen. (b) Immediate postoperative intraoral view of the surgical site.

## Discussion

Maxillary ameloblastomas warrant particular attention because their clinical behaviour is influenced by local anatomy. The relatively thin cortical plates and cancellous architecture of the maxilla, together with proximity to the maxillary sinus, nasal cavity, orbit, and skull base, can permit tumour extension before prominent extraoral changes become apparent. This anatomical setting helps explain why a maxillary lesion may already show cortical destruction, tooth displacement, root resorption, or involvement of adjacent structures at the time of diagnosis [1-3,5,8,9].

The acanthomatous pattern is defined histologically by squamous metaplasia and keratin formation within the central portions of odontogenic epithelial islands. In this case, these microscopic findings, together with peripheral palisading and reverse nuclear polarity, established the diagnosis and helped distinguish the lesion from other entities with overlapping clinical or radiographic appearances.

In the present case, the lesion involved the anterior maxilla, crossed the midline, and caused cortical perforation, external root resorption, and nasopalatine canal involvement. These findings demonstrate its locally aggressive behaviour despite its benign histopathological nature. The radiographic features were consistent with those previously reported for aggressive maxillary ameloblastomas, including expansile osteolytic destruction, cortical expansion and perforation, tooth displacement, and external root resorption [3,4,8,10,11].

These features are not pathognomonic and may overlap with those of odontogenic myxoma, odontogenic keratocyst, central giant cell granuloma, aneurysmal bone cyst, and other multilocular jaw lesions. Imaging alone may therefore be insufficient for definitive diagnosis. Compared with panoramic radiography, CBCT provided high-resolution three-dimensional visualization of the lesion and accurately demonstrated its extent, cortical perforation, root resorption, tooth displacement, and nasopalatine canal involvement. These findings were valuable for surgical planning and highlight the role of CBCT in evaluating locally aggressive maxillary lesions [6,8,11,12].

Complete surgical excision is the preferred treatment for solid and multicystic ameloblastomas because conservative procedures, such as enucleation and curettage, are associated with higher recurrence rates. Owing to the infiltrative growth pattern of ameloblastoma, tumour cells may extend beyond the apparent radiographic margins. Complete resection with adequate surgical margins is therefore necessary to reduce the risk of recurrence [5,9,11,13].

Partial maxillectomy was selected in this case because of the lesion’s extensive local invasion and cortical perforation. Histopathological examination of the resected specimen confirmed the preoperative diagnosis, and postoperative healing was uneventful. Long-term surveillance is essential because ameloblastoma may recur several years after treatment, particularly in the maxilla, where complete excision may be difficult. Regular clinical and radiographic assessment, with CBCT when indicated, may facilitate early detection of recurrence. No clinical or radiographic evidence of recurrence was detected during the 2-year follow-up period [5,8,9,11,14].

The principal strength of this report is the comprehensive correlation of the clinical, radiographic, and histopathological findings in a rare acanthomatous ameloblastoma of the anterior maxilla that crossed the midline. The case demonstrates the complementary roles of clinical examination, CBCT, and histopathological evaluation in establishing an accurate diagnosis and guiding surgical management.

The principal limitation is that this is a single case, so the findings should not be generalised to all patients with acanthomatous ameloblastoma or to other multilocular maxillary lesions. Molecular investigations, including assessment of MAPK (mitogen-activated protein kinase) pathway alterations such as the BRAF V600E mutation, were not performed. In addition, the exact duration of symptoms and dates of all interim postoperative visits were not available in the clinical record. Although no recurrence was detected at 24 months, this interval is insufficient to exclude late recurrence, and continued surveillance is required. Nevertheless, the case adds to the limited literature on this uncommon histopathological variant and demonstrates the value of clinicoradiographic-pathological correlation [10,15-17].

Postoperative care

Postoperative management included analgesics, antibiotics, oral-hygiene instructions, nutritional support, and scheduled clinical reviews. The patient was counselled regarding the risk of local recurrence and the need for long-term surveillance. The postoperative course was uneventful, with satisfactory healing of the surgical site and gradual improvement in oral function. The available record documents clinical and radiographic assessment at 24 months; exact dates of all interim visits were not available.

Follow-up and outcomes

No immediate postoperative complications were observed. At the documented 24-month follow-up, clinical and radiographic examinations demonstrated satisfactory healing and no evidence of recurrence (Figure 5a-c). This two-year disease-free interval is reassuring but does not exclude late recurrence. Because maxillary ameloblastoma can recur after a prolonged interval, continued long-term clinical and radiographic surveillance was advised.

**Figure 5:**
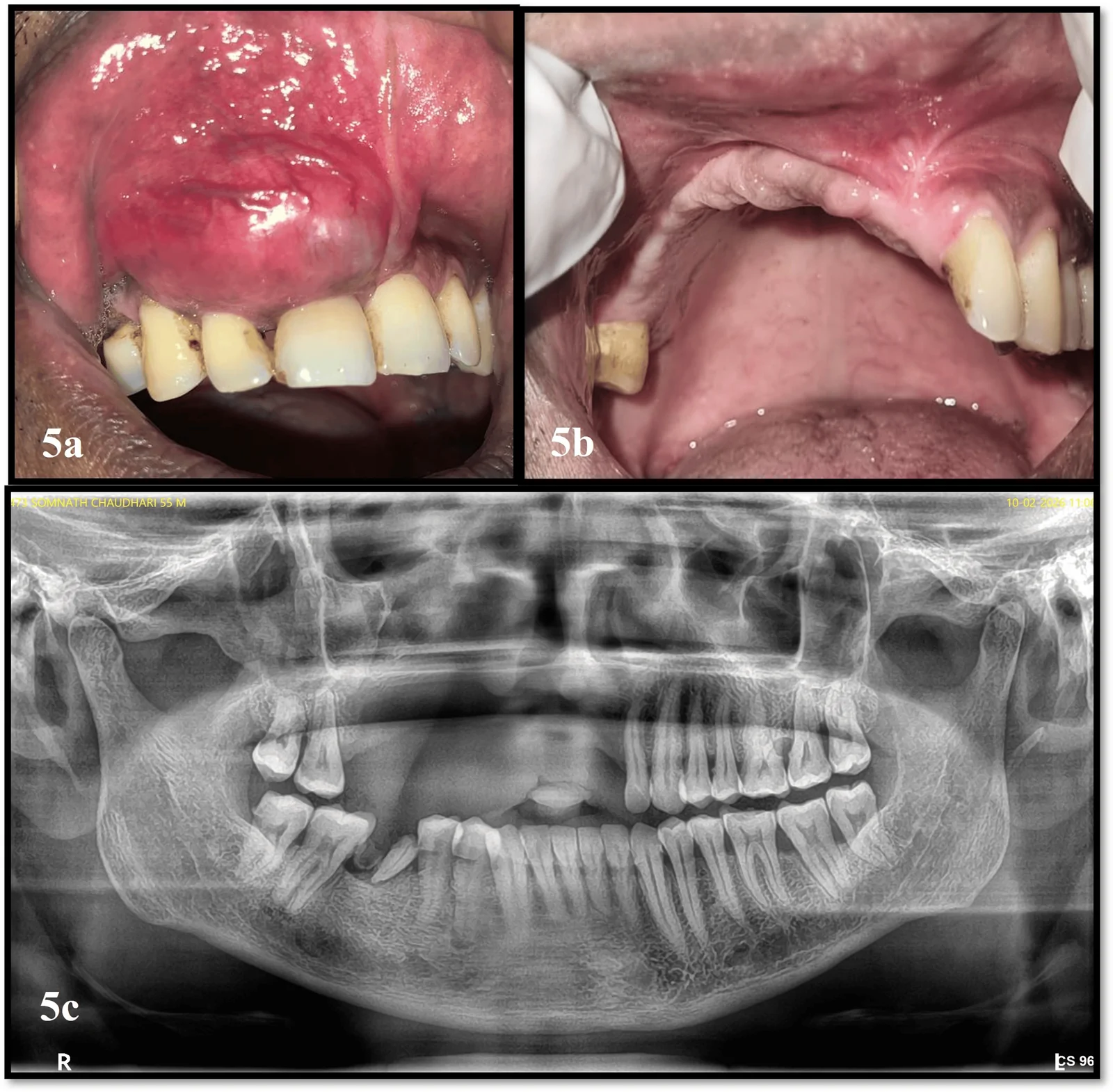
(a) Preoperative intraoral view. (b) Intraoral view after healing. (c) Panoramic radiograph obtained 2 years after surgery, showing no evidence of recurrence.

Timeline

The key events in the patient’s clinical course are summarised in Table 1. 

**Table 1:** Timeline of the patient’s clinical course. CBCT: cone-beam computed tomography.

Time point	Clinical event
Initial presentation	Progressive swelling in the anterior maxilla accompanied by pain during mastication
Clinical examination	Diffuse anterior maxillary swelling crossing the midline, with cortical expansion, vestibular obliteration, and eggshell crackling
Panoramic radiography	Mixed radiolucent-radiopaque lesion with displacement of adjacent teeth and external root resorption
CBCT examination	Multilocular expansile lesion crossing the midline, with cortical perforation, root resorption, tooth displacement, and nasopalatine canal involvement
Incisional biopsy	Histopathological diagnosis of acanthomatous ameloblastoma
Treatment	Partial maxillectomy with complete surgical excision
Histopathological confirmation	Diagnosis of acanthomatous ameloblastoma confirmed in the resected specimen
Postoperative follow-up	Uneventful healing with no clinical or radiographic evidence of recurrence during two years of follow-up; long-term surveillance advised

## Conclusions

This case demonstrates that acanthomatous ameloblastoma can present as a locally destructive anterior maxillary lesion despite benign histopathology. In this patient, the lesion crossed the midline and was associated with cortical perforation, external root resorption, tooth displacement, and nasopalatine canal involvement. CBCT defined the three-dimensional extent of disease, while histopathological examination established the definitive diagnosis. Partial maxillectomy achieved complete surgical excision, and no clinical or radiographic recurrence was identified at 24 months. These observations are limited to a single case, and the two-year follow-up does not exclude late recurrence; prolonged surveillance remains necessary.
